# Gut-derived hemodynamic echo loops: microbial electrogenesis as a hidden driver of refractory septic shock

**DOI:** 10.1097/MS9.0000000000004634

**Published:** 2025-12-19

**Authors:** Ureba Memon, Dua Memon, Mahnoor Fatima

**Affiliations:** a Department of Medicine, Liaquat University of Medical & Health Sciences, Jamshoro, Pakistan; b Department of Medicine, Shaheed Ziaur Rahman Medical College, Rajshahi Medical University, Bogura, Bangladesh

*To the Editor,*

Refractory septic shock is a major issue in critical care, which presents itself as incomplete vasoplegia that does not respond to sufficient fluid resuscitation and maximum doses of vasopressor. Conventional paradigms, including cytokine spikes, nitric oxide imbalance, and mitochondrial injury, cannot fully explain the irreversible hemodynamic breakdown in some patients. More recent findings suggest human-related intestinal microbes can undergo extracellular electron transfer (EET) during anaerobic fermentation. This finding verifies the hypothesis that microbial electrogenesis could be an unrecognized shock physiology pathway, potentially interacting with host vascular signaling^[1,2]^.

The human gut isolates, such as *Enterococcus, Klebsiella*, and *Citrobacter* species, have been shown to electrogenically behave through membrane-bound redox systems, capable of transferring electrons to external acceptors extracellularly^[1,3]^. Such EET pathways can alter gut redox conditions and also regulate epithelial and endothelial signaling, as well as local immune recruitment^[4]^. Those bioelectrical interactions offer a mechanistic story of hemodynamic echo-loops. By “hemodynamic echo loops,” we refer to a cycle in which microbial redox activity alters vascular tone, which then modifies perfusion patterns and reinforces the same dysfunction. That is, electrogenic microbes increase their own survival by eliciting microvascular responses that augment local perfusion and nutrient availability.

Among these electrogenic organisms, *Enterococcus faecalis* has been specifically well characterized.In *E. fecalis* reduced demethylmenaquinone in the cytoplasmic membrane is also necessary in EET, but heme proteins are not involved. The activity of cytochrome bd oxidase suppresses EET, providing critical information about bacterial EET processes as well as the design of electrochemical systems in microbes^[2]^. Additionally, other human gut isolates conduct extracellular electron transfer, as demonstrated by electrochemical analyses, highlighting their bioelectrochemical activity in the gut environment^[3]^. Microbial extracellular electron transfer in the mammalian gut influences host redox balance and mucosal immune cell recruitment, potentially impacting vascular and immune functions, as demonstrated by electrochemical analyses of gut bacteria^[5]^. This suggests a role in a broader systemic sphere when such organisms translocate or proliferate in dysbiosis, such as in sepsis.

Electrogenic bacteria utilize electrons extracellularly to external acceptors and affect their immediate surroundings through metabolism^[4]^. This interaction may form feedback loops in microenvironments, although there is currently no evidence of direct links between this and increased perfusion or worsening vasoplegia, and it is thus still a hypothesis.

Microbial EET is currently being identified in a wide variety of environments, and novel approaches to identifying microbial electron shuttles are under development^[6]^. Though yet to be clinically studied, these tools conceptually are useful in supporting future diagnostic innovation in sepsis.

Altogether, microbial electrogenesis is a biologically plausible and increasingly researched cause of the persistent vasoplegia of the refractory septic shock. The discovery of gut microbes that have a direct impact on the vascular tone of their hosts through the redox-mediated transfer of electrons redefines septic shock as both an immunologic crisis and a possible gut–vascular electrical disease. Integrating microbial electrobiology with the conventional science in hemodynamics can offer an entirely novel frontier in diagnostic activity, in which redox biomarkers, microbial electroflux indicators, or gut-derived electroactive metabolites could be employed to detect patients at risk of nonresponsiveness to shock. Interestingly, this opinion also highlights a potential treatment approach: regulating intestinal microbial electron transfer to reinitiate vascular responsiveness. The conceptual breakthrough that can improve outcomes is the investigation into this gut–vascular electrogenic axis, as the proportion of refractory septic shock remains unacceptably high.

This is in line with the TITAN Guidelines on the need for transparency in AI use in healthcare^[7]^.

## Data Availability

Not applicable to this study.
